# Can dietary fibre help provide safer food products for sufferers of gluten intolerance? A well-established biophysical probe may help towards providing an answer

**DOI:** 10.1186/2046-1682-5-10

**Published:** 2012-05-17

**Authors:** M Samil Kök, Richard Gillis, Shirley Ang, David Lafond, Arthur S Tatham, Gary Adams, Stephen E Harding

**Affiliations:** 1 Department of Food Engineering, Abant Izzet Baysal University, 14280, Bolu, Turkey; 2 National Centre for Macromolecular Hydrodynamics, University of Nottingham, Nottingham, LE12 5RD, UK; 3 Kellogg Company, Battle Creek, MI, 49017-3517, USA; 4 Cardiff Metropolitan University, Cardiff School of Health Sciences, Cardiff, CF5 2YB, UK; 5 University of Nottingham Institute of Clinical Research, Queens Medical Centre, Nottingham, NG 7 2UH, UK

**Keywords:** Gluten intolerance, Dietary fibres, Protein-polysaccharide interactions, T-cell response

## Abstract

Gluten intolerance is a condition which affects an increasing percentage of the world’s population and for which the only current treatment is a restrictive gluten free diet. However could the inclusion of a particular polysaccharide, or blends of different types, help with the provision of ‘safer’ foods for those individuals who suffer from this condition? We review the current knowledge on the prevalence, clinical symptoms and treatment of gluten intolerance, and the use and properties of the allergens responsible. We consider the potential for dietary fibre polysaccharides to sequester peptides that are responsible for activation of the disease in susceptible individuals, and consider the potential of co-sedimentation in the analytical ultracentrifuge as a molecular probe for finding interactions strong enough to be considered as useful.

## Introduction

There is growing interest in the use of traditional food-type of large carbohydrate molecules such as galactomannans, glucomannans and arabinoxylans for therapeutic biopharmaceutical purposes ranging from blood plasma substitutes to mucoadhesive drug delivery systems. There has been a suggestion that these molecules may also offer a protective role for the mucosal epithelia for sufferers of gluten protein intolerance, by interacting with the gluten proteins. A well established biophysical technique – sedimentation velocity in the analytical ultracentrifuge – may provide an answer to the important question as to whether these interactions would be strong enough for gluten proteins passing through the gastrointestinal tract.

### Gluten intolerance

**Figure 1:**
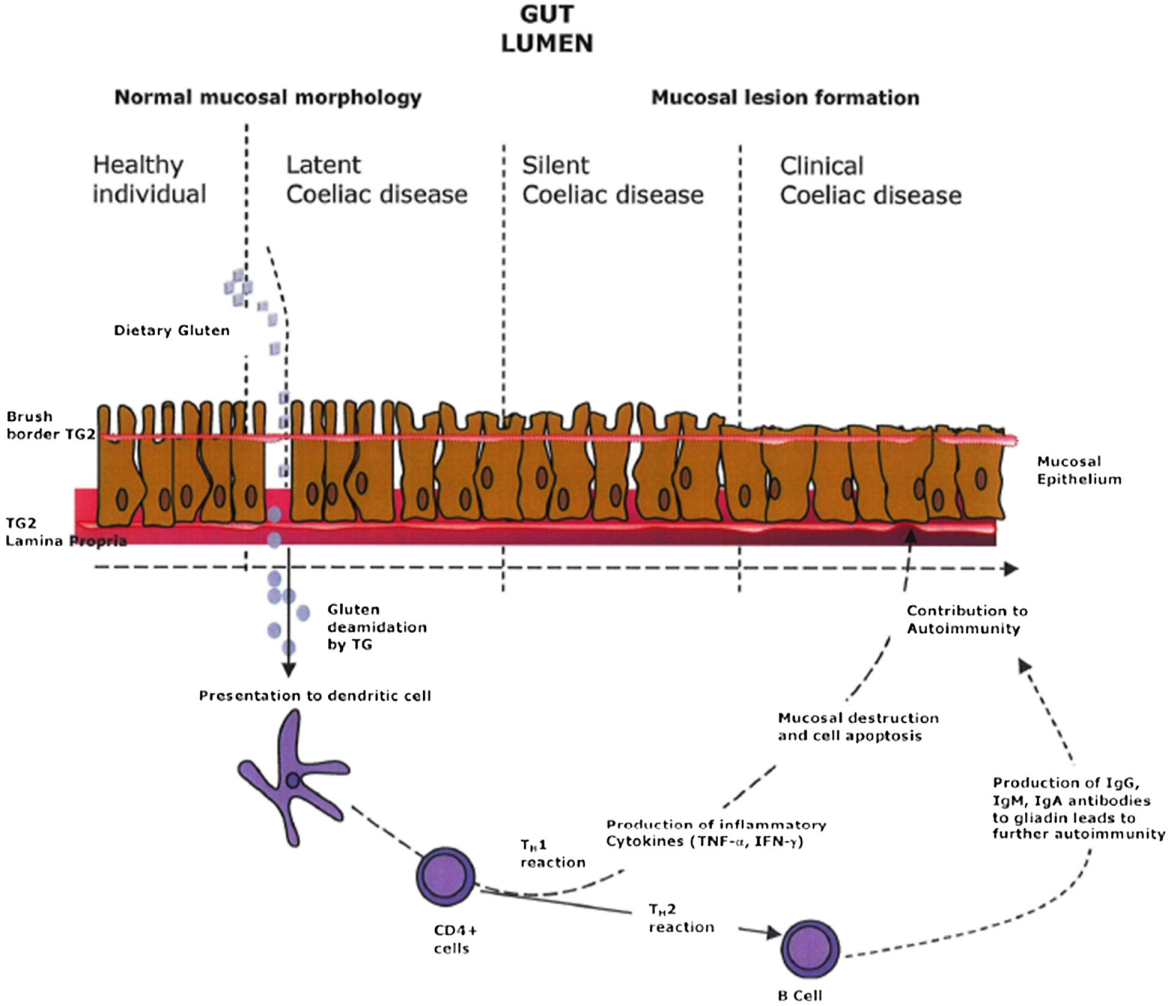
Prolamin derived peptides interacting with the mucosal epithelia of the small intestine of a sufferer of coeliac disease initiating an IgE mediated response.

Gluten intolerance is a T-cell mediated autoimmune condition (as distinct from an allergic IgE mediated immune response) of the small intestine that occurs when an individual with a genetic predisposition to the condition ingests the proteins of wheat, barley and rye, and possibly oats [1]. The ingestion of gluten and related proteins leads to damage of the mucosal lining and the flattening of the villi of the small intestine (Figure 1) resulting in the malabsorption of nutrients from the diet. The condition is permanent, and damage to the small intestine will occur every time gluten is consumed, regardless of whether symptoms are present or not [2], the only current treatment is a total exclusion of gluten and related proteins from the diet – a gluten free diet.

The condition has been recognised for many centuries, but what is considered as the first detailed description was given by Dr Samuel Gee in 1887 and described as a malabsorption of ingested food in children: removal of wheat flour and wheat products from the diet was later seen to alleviate symptoms associated with the disease [3]. Van de Kamer and Weijers [4] found that the gliadin fraction from wheat was active in patients with gluten intolerance. Equivalent fractions from rye, barley and possibly oats were also considered coeliac active and this activity could not be removed by digestion with pepsin, trypsin or pancreatin. Therefore, foods that contain the proteins of wheat, barley, rye, oats (possibly) and the relatives of wheat (e.g. triticale and kamut), beverages containing malted grains and any processed foods that contain these as ingredients must be excluded from the diet of coeliac patients.

The clinical symptoms associated with untreated disease are varied and can lead to delays in diagnosis. Symptoms vary from fatigue, headaches, abdominal complaints, diarrhoea, joint complaints to vitamin (both fat and water soluble) and mineral deficiencies, which can lead to anaemia (iron and folate) and hypocalcaemia [5]. An increased risk of gastrointestinal malignancy is associated with undiagnosed or inadequately managed gluten intolerance [6]. The disease is also associated with other autoimmune diseases (type-I diabetes, autoimmune thyroid and liver disease and inflammatory bowel disease), osteoporosis, neurological disturbances and growth disturbances [6].

### Prevalence of gluten intolerance

Over the past two decades, the perception of gluten intolerance has transformed from the concept of a rare disease affecting primarily children of northern European ancestry with gastrointestinal symptoms, to a very common condition of people of all ages worldwide. Indeed the condition has recently received high profile coverage in the media following the improved performances of top sports stars after moving to gluten-free diets [7]. Recent studies have indicated that the condition is not confined to those of Western countries or those of Northern European descent, where the incidence of the disease approaches 1%, but is as common in the Middle East [8]. The condition is under-diagnosed due to a number of factors. Often individuals display only mild or subclinical symptoms, and until the recent introduction of serological tests diagnosis depended on determining changes in intestinal histology (which is still the standard method). More than 60% of newly diagnosed patients are adults, with 15–20% being over 60 years of age [5].

From the above studies it is evident that within populations genetic factors are very strong determinants of gluten intolerance, with the major risk attributed to the specific genetic markers known as HLA-DQ2 and HLA-DQ8 that are present in affected individuals. The gluten proteins of wheat, barley and rye interact with these HLA molecules and activate the abnormal intestinal response. However, gluten intolerance develops only in a minority of DQ2 and DQ8 positive individuals and other environmental factors are implicated, such as early weaning onto solid food, breast feeding and gastrointestinal infection [9].

### Control of gluten intolerance

The only known effective treatment for gluten intolerance is a life-long gluten-free diet (GFD). There are few systematic studies in the literature on the factors affecting an individual’s ability to adhere to a GFD but a number of factors have been identified. These include compliance, particularly among adolescents, where dietary diaries indicate compliance levels between 50–95%, however, serological/intestinal biopsy studies on the same subjects indicate different degrees of intestinal damage [10]. Poor product information is another contributing factor relating to the gluten content of foods and the fact that gluten products can be ‘hidden’ in foods where they would not be expected to form part of a particular product. Individuals differ in their sensitivity to gluten so that an activating dose of gluten for one individual may not elicit a response in another [11]. The availability and price of gluten free (GF) foods is another factor, often there are limited ranges of GF food products available and these are considerably more expensive than conventional products and can place an economic burden on the individual and their family. The conclusion is that in patients attempting to adhere to a GFD, mucosal damage can occur from the ingestion of gluten due to a number of factors that may be outside the control of the individual.

There is also a problem with the acceptability to consumers of GF products. The unique properties of wheat gluten make it difficult to replace and currently many GF products available on the market are of low attraction, exhibiting poor mouth feel and flavour. The use of starches, gums and hydrocolloids represent the most widespread approach used to mimic gluten in the manufacture of GF bakery products, due to their structure-building and water binding properties. Novel approaches including the application of dietary fibres and alternative protein sources combined with response surface methodology are also emerging [12]. Preparation of GF pasta is also difficult, as the gluten contributes to a strong protein network that prevents dissolution of the pasta during cooking. The diversification of GF raw materials which can be used may also cccprocesses [13].

GF foods can be prepared from gluten containing ingredients, where the gluten component has been removed. In the USA and Canada food labelled GF must be devoid of wheat whereas in Europe products labelled as “gluten-free” are permitted to contain wheat starch [12]. The threshold amounts of gluten that activate gluten intolerance have produced conflicting results and it has been concluded that it is the total amount of gluten ingested over time rather than the concentration of gluten in the food product that is important. It is recommended that the ingestion of gluten should be kept at less than 50 mg gluten per day in the treatment of gluten intolerance [14]. The recently revised recommendations of the WHO/FAO [15] indicate that products only be called ‘gluten free’ if there is less than 20 ppm of gluten in the finished product. In Europe new legislation requires that products labelled ‘gluten free’ (usually made from foods that do not naturally contain gluten) must contain less than 20 ppm gluten. Foods that have been treated to reduce gluten content and contain between 20 and 100 ppm are to be labelled “very low gluten” [15]. However, individuals differ in their sensitivity to gluten and even these low levels may be sufficient to cause intestinal damage in some individuals. ‘Gluten-free’ foods themselves can be contaminated by gluten containing cereals, for example in one study on four flour samples and thirteen brands of biscuit, two flour samples and one brand of biscuit tested positive for gluten contamination [16].

Whereas untreated coeliac disease can result in inadequate nutrition for the individual, there is evidence that strict adherence to a GFD can also result in nutritional inadequacies. Few gluten-free products are enriched or fortified, adding to the risk of nutrient deficiencies. Poor vitamin status has been reported for 50% of patients adhering to GFD for 10 years, an increased incidence of obesity and poor nutrient intakes [17].

### The structure of wheat gluten

Wheat gluten is defined as the proteinaceous cohesive mass that remains when dough is washed to remove starch and has the unique properties (among the cereals) of elasticity and viscous flow, properties associated with the prolamins, the seed storage proteins. The prolamins are unusual in that they are soluble in aqueous alcohols, their amino acid compositions are rich in glutamine and proline (combined 25–60 mol%) and their molecular weights (molar masses) vary from about 30,000 to 100,000 Daltons (g/mol).

**Figure 2:**
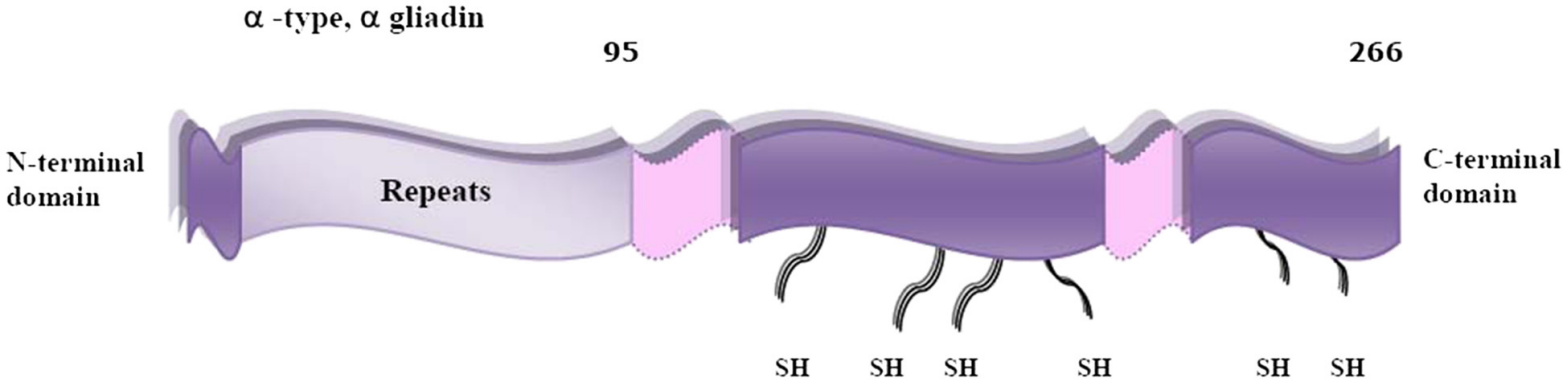
**Structure of typical a S- rich prolamin, α-gliadin.** The domains consist of a short non-repetitive N-terminal domain, a repetitive domain (that contains the majority of the coeliac active pitopes), a glutamine-rich domain, followed by a non-repetitive domain, a glutamine-rich domain and a C-terminal non-repetitive domain [18,19].

**Figure 3:**
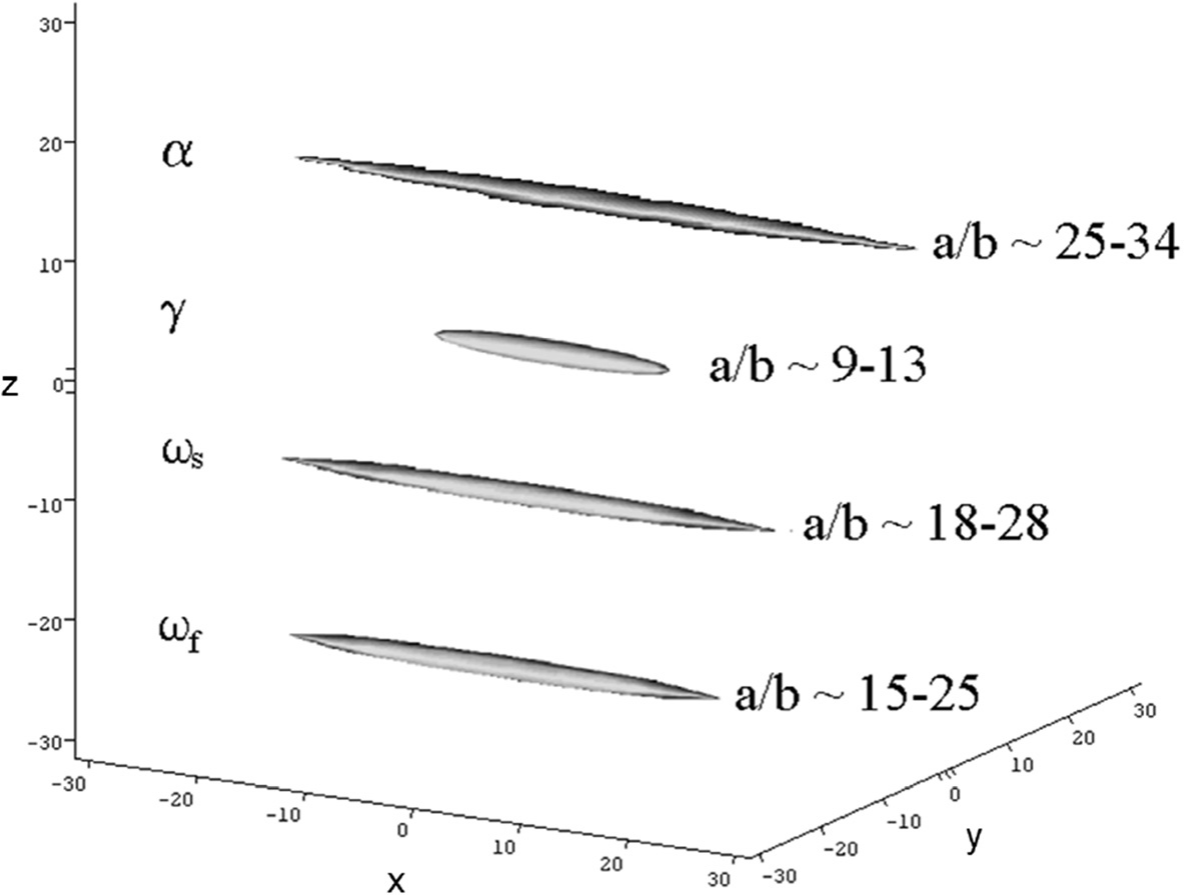
**Axial ratio determinations of the principal subfractions of α−, γ− and ω−gliadins in 70% aqueous ethanol solutions.** The principal semi-axes a,b,c (with a > b and c = b for a prolate ellipsoid) are drawn in the direction of the orthogonal Cartesian axes x,y,z. Reprinted, with permission from Springer, from [20].

The prolamins can be divided into two groups on the basis of their solubility characteristics, namely gliadins which are soluble in aqueous alcohols (and unless digested with enzymes only sparingly soluble in aqueous systems) and glutenins which are only soluble on the addition of reducing agents. Gliadins are further divided into sulphur-poor and sulphur-rich on the basis of their sequences. The S-poor prolamins are rich in glutamine (40–50 mol%), proline (20–30 mol%) and phenylalanine (7–9 mol%) and consist almost entirely of repeated sequences containing no cysteine residues for covalent cross-linking. The S-rich prolamins (Figure 2) are the major group of prolamins and account for about 80% of the total fraction [18,19]. They comprise the α- and γ-type gliadins, which are monomeric with intramolecular disulphide bonds and the low molecular weight (LMW) subunits of glutenin of wheat, which contain both intra- and intermolecular disulphide bonds. A recent study of the heterogeneity and conformation in solution of gliadin proteins from wheat shows several clearly resolved components [20]. All the proteins are shown to be extended molecules with axial ratios ranging from approximately 10 to 30 (Figure 3) with the α-types appearing the most extended and γ- the least. In Figure 3 although only one structure is shown for each of the α− and γ− gliadins, each of these is the average of several subfractions (Table 1).

**Table 1:** Heterogeneity of the α− and γ−gliadins in wheat: their sedimentation coefficients and relative abundance

**gliadin fraction**	**gliadin subfraction**	**s**^**o**^_**20,w **_**(S)**	**proportion in fraction**
α	α_0.8_	0.80±0.05	62%
	α_1.9_	1.90±0.05	18%
	α_2.5_	2.50±0.05	20%
γ	γ_1.2_	1.20±0.10	83%
	γ_2.8_	2.80±0.05	12%
	γ_4.6_	4.60±0.13	5%

Adapted, with permission of Springer, from [20].

**Figure 4:**
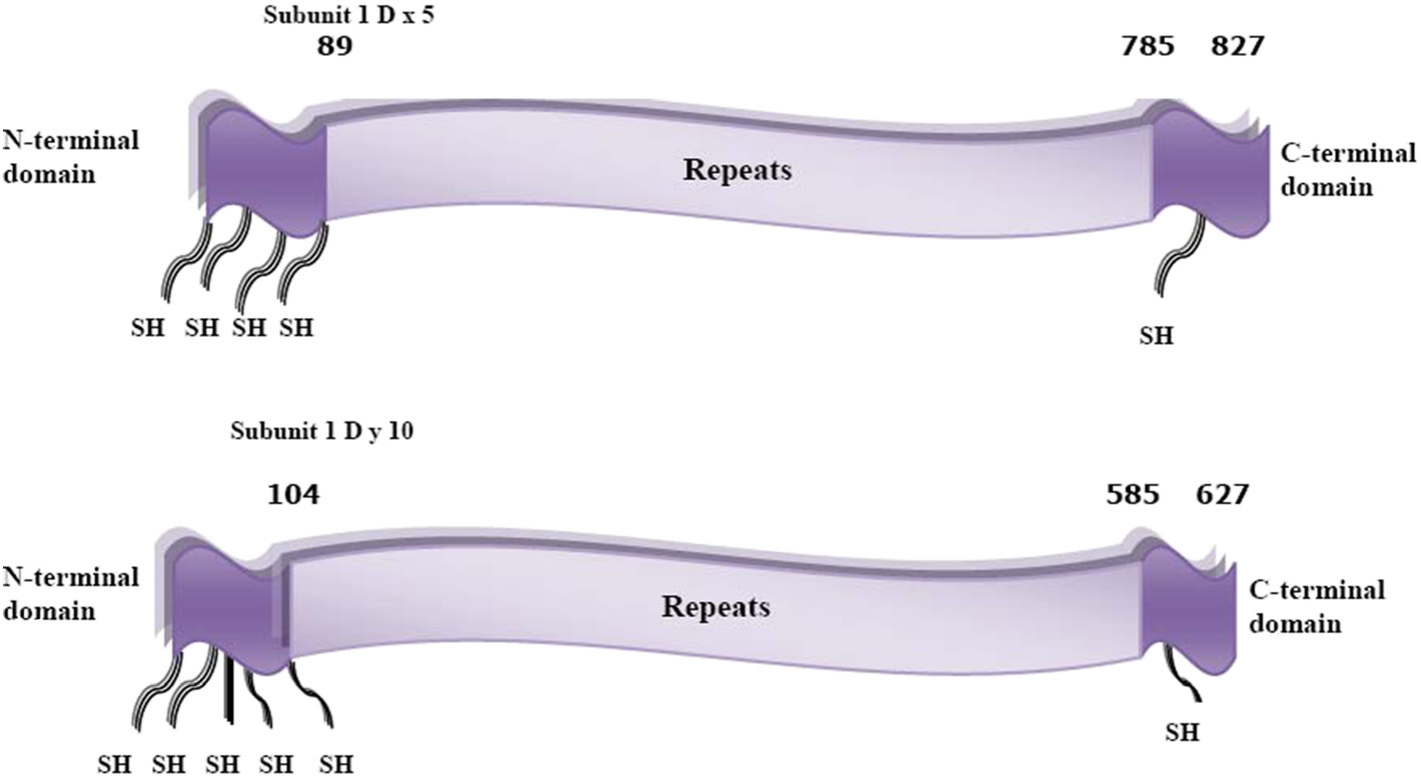
**Schematic structure of x and y type high molecular weight (HMW) subunits.** The x- and y-types have 80% similarity in structure. A large central domain composed of repeating amino acid sequences rich in glutamine and proline, flanked by N- and C-terminal domains made up of non-repetitive sequences that contain highly conserved cysteine residues [21,22].

This group of proteins consists of two structural domains, a repetitive N-terminal and non-repetitive C-terminal domain. The N-terminal domain consists of proline and glutamine-rich repeated sequences based on PQQX, PQQPFPQ, PQQQPFPS and PQQPX(X). The C-terminal domain consists of non-repetitive sequences and contains most or all of the cysteine residues. The high molecular weight (HMW) subunits of wheat consist of three domains (Figure 4), namely non-repetitive N- and C-terminal domains with a large repetitive central domain consisting of PGQGQQ, GYYPTSPQQ, GYYPTSLQQ and in some GQQ repeated sequences [21,22]. This group of proteins contributes to the elastic nature of gluten. The dominant feature of all of the prolamins is blocks of repeated sequences and it is specific parts of these that bind to T-cells and activate a response from receptors in the mucosal epithelia of sufferers of coeliac disease.

Upon exposure to gliadin, and specifically to peptides found in prolamins, the enzyme tissue transglutaminase modifies the protein and the immune system cross-reacts with the small-bowel tissue, causing an inflammatory reaction. There is evidence that substitution of deamidated glutamine residues at a critical position along the gliadin sequence dramatically changes immunological activation. Alanine substitution at position P38 of sequence 3 l-49 of α-gliadin, was found to result in an increased DQ2-binding affinity but also in loss of toxicity. The toxicity of many gluten epitopes has thus far been investigated, although the region 57–75 of α-gliadin remains the most studied [23].

Patients with coeliac disease recognise peptides derived from each of the subfractions S-rich, S-poor and HMW subunits and homologous sequences in rye secalins and barley hordeins. Characterised wheat gluten T-cell determinants include the peptides PFPQPELPY, PQPELPYPQ, EGSFQPSQE, EQPQQPFPE which require the deamidation of a single glutamine residue (underlined) for optimal activity, whereas the HMW derived sequence QGYYPTSPQ does not [24-26]. The characteristics of these peptides are that they are highly protease resistant and proline-rich. It is this group of peptides/proteins containing these reactive sequences that need to be removed from foods and/or screened from the mucosa to render them safe for consumption by coeliac patients.

More recent research has shown that modification of gluten by binding of the amino acid methionine, preserved the functionality of gluten but gave a reduced reactivity to serum IgA from gluten intolerance patients [27]. However rather than working to permanently modify the structure of gluten through genetically modifying wheat it would be better if a more environmentally and socially acceptable solution could be found.

### Use of dietary fibre (DF) polysaccharides

It would be very useful if people who suffer from gluten intolerance could consume a limited number of low gluten based products without suffering from the consequence, or if the trace amounts of gluten in “gluten free” foods (which can still cause severe problems) could be taken out by another non-digestible food ingredient. To achieve this would mean preventing coeliac activating peptides from coming into contact with the mucosal epithelia and its receptors. Could the addition of a natural ingredient or combination of ingredients be the answer?

**Figure 5:**
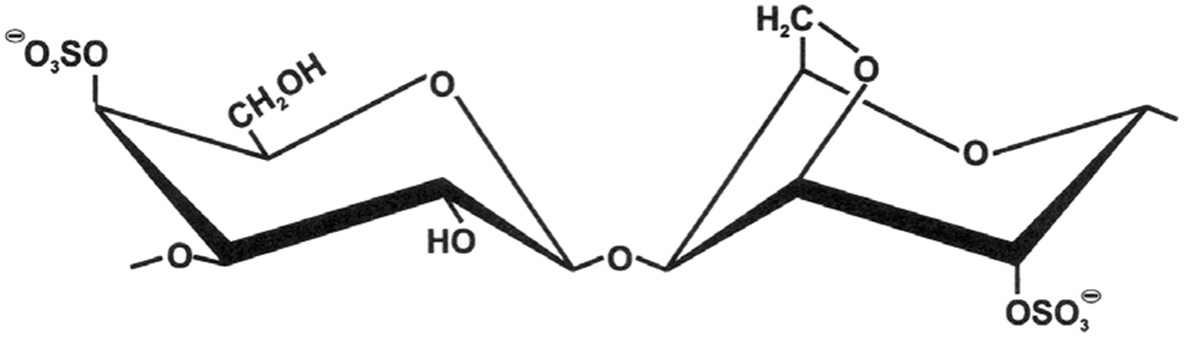
**Disaccharide repeat structure of iota-carrageenan.** It is an alternating repeat structure of β-D-galactose sulphate linked (1–4) to 3,6 anhydro-α-D-galactose with the anhydro-galactose residue sulphonated at carbon position 2.

A particular group of complex carbohydrate substances which are used as dietary fibre may hold the key here. Dietary fibre carbohydrates (Figure 5), sometimes referred to as “non-digestible carbohydrate” or NDC, are all essentially polysaccharides and associated lignins in the diet that are not digested by the endogenous secretions of the human digestive tract and are of considerable physiological importance [28]. They influence the digestion of food in general and in particular reduce the insulin needs of people with diabetes, influence bile acid metabolism, alter lipid digestion, cholesterol absorption and protect against colonic cancer [29]. Byrnes et al. [30] found that meals which included bread containing partially depolymerized guar galactomannan, gave a reduction in postprandial insulin resistance in healthy middle aged men at risk of coronary heart disease. Addition of partially hydrolyzed guar gum to the diet reduced laxative dependence in a nursing home population. It also reduced the incidence of diarrhoea in septic patients receiving total enteral nutrition, reduced symptoms of irritable bowel syndrome and increased production of *Bifidobacterium* in the gut [31].

Another class of undigestible polysaccharide being used in health products is chitosan. This is a solubilised form of chitin – from the shells of crabs, lobsters, crustaceans and also from some types of mushroom. What distinguishes it from many other polysaccharides is that whilst most others are either polyanionic (negatively charged) or neutral (no charge), chitosans are polycationic (positively charged) and appear to be ideal bioadhesive materials [32].

### Potential of protein-polysaccharide interactions

It is known from the work of Tolstuguzov and others that some combinations of proteins and polysaccharides can form complexes [33]. Proteins can also self-associate by themselves strongly and weakly [34] and polysaccharides can form strong self-aggregation complexes by themselves and also with other macromolecules such as mucins, forming the basis of mucoadhesive strategies [35]. Very recently one class of polysaccharide has been shown by the powerful method of sedimentation velocity in the analytical ultracentrifuge to oligomerize in a way more reminiscent of proteins [36].

**Figure 6:**
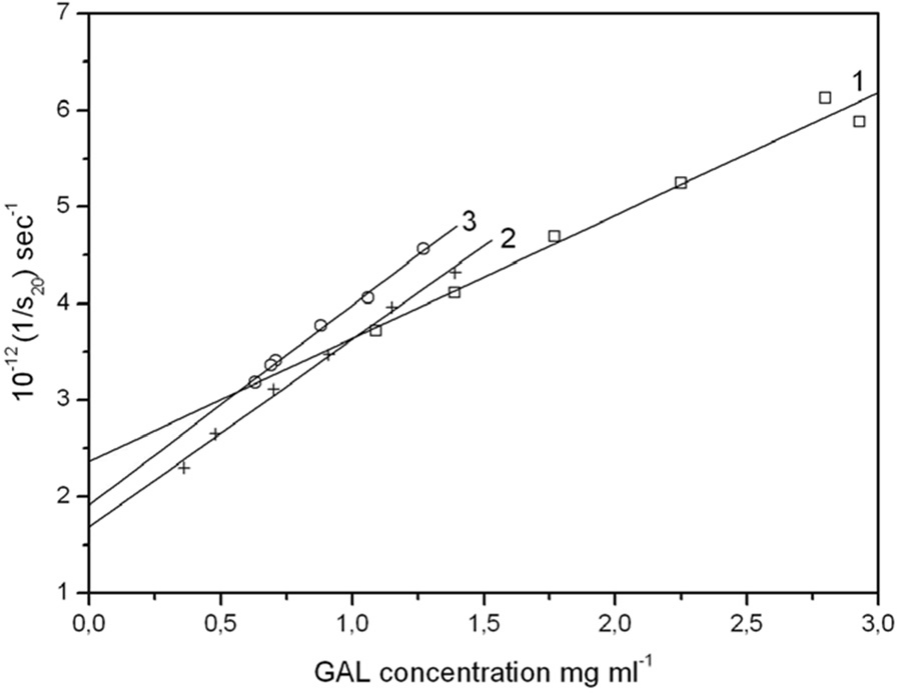
**Sedimentation velocity isotherms (sedimentation coefficient versus concentration plots) for mixtures of locust bean gum and pepsin-trypsin gliadin (PT-gliadin) digests**[37]. Solid squares are for the control solutions of locust bean gum (fitted line 1). Plus symbols correspond to PT-gliadin: galactomannan mixture with a PT-gliadin mixture ratio of ~4:1 (fitted line 2) and the circles (fitted line 3) with the ratio ~ 1:4. Both sets of mixtures show significantly higher extrapolated s_20_^o^ values and higher concentration dependencies of s_20_, consistent with a significant interaction. Reprinted with permission of Elsevier.

With regards to interactions of seed storage proteins with polysaccharides this is a surprisingly underexplored area considering the extent of the health problems associated with these proteins, although some rheological studies have suggested an interaction with cellulose derivatives [37]. One earlier study [38] focused on the interactions of pepsin-trypsin digested gliadin preparations with locust bean gum, using analytical ultracentrifugation as the principal probe. Evidence of an interaction was seen based on comparisons of the sedimentation coefficients *s*^*o*^_20_ and concentration dependence regression coefficients *k*_s_ for mixtures and reactants (Figure 6).

The wide spectrum of functional properties associated with different polysaccharides can be explained in terms of differences in conformation, size, or solubility of these polymers [39]. There is evidence to suggest that the potential of some to interact with protein could protect sensitive persons from harmful allergic reactions involving wheat, soya and milk proteins [40]). Synthetic polymers have been shown to interact with gliadins and suppress gliadin induced toxicity in intestinal epithelium in a mouse model [41]: it is reasonable to suppose therefore that natural polysaccharides may show similar properties.

### Detecting interactions and assaying the interaction strength using the analytical ultracentrifuge

The study of Seifert et al. [37] was based on measurements performed in a classical Beckman Model E ultracentrifuge with Schlieren optics. Since then there have been considerable advances in the methodology – the use of the new generation analytical ultracentrifuge with on-line data capture of optical records of the changing concentration distribution in an ultracentrifuge cell – using both UV-absorption optics and refractometric optics - together with advances in software facilitating the almost routine measurement of distributions of sedimentation coefficient.

**Figure 7:**
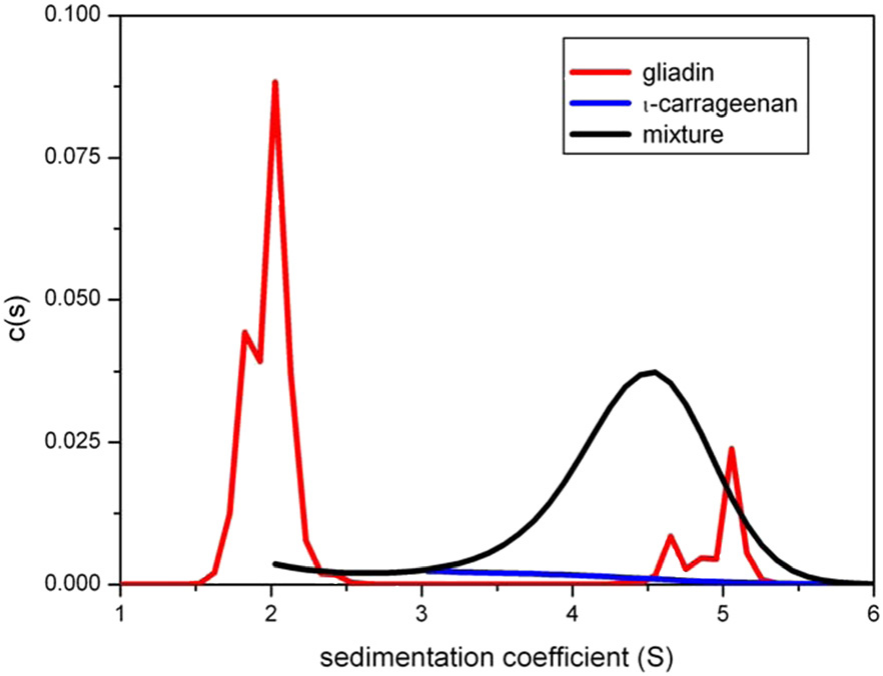
**Sedimentation coefficient distribution diagrams of gliadins and iota carrageenan in aqueous phosphate-chloride buffer.** c(*s*) = the population of species with a sedimentation coefficient between *s* and d*s*. UV-absorption optics at 280 nm were used showing only the gliadins – and whatever they may have interacted with. Red line: gliadin only control at 5.0 mg/ml loading concentration showing material sedimenting at 2 S and a small amount of aggregated material at ~ 5 S. Blue line *i*- carrageenan control at 1.0 mg/ml: the sedimenting material is almost transparent at 280 nm. Black line (same concentrations) – mixture showing a substantial amount of material sedimenting at ~ 4.5 S: this may indicate an interaction with gliadin.

The dual on-line detection system of UV-absorption and refractometry on the Beckman XL-I ultracentrifuge - which has now fully superseded the old Model E’s - facilitates the measurement of co-sedimentation as an assay for interaction [42]: gliadin proteins tend to have low sedimentation coefficients (~1–2 S) [20] and show strong UV absorbance at 280 nm whereas most polysaccharides do not. Hence polysaccharides – which tend to sediment >1 S - are almost “invisible” in mixtures at 280 nm unless gliadin has bound to them. In this way an interaction appears to have been observed for example between iota-carrageenan and gliadin in dilute aqueous solution (Figure 7). [43].

### Concluding remarks

Although promising, the goal now is to see if there exists a non-toxic biopolymer combination providing not only a strong interaction with the form that gliadins present themselves to mucosal epithelia – the pepsin-trypsin digested form - but an interaction which will withstand the physiological stresses in the alimentary tract and the bioprocessing stresses during food preparation. The value of the ultracentrifuge as an assay procedure is it involves no columns or membranes – as required by chromatographic or field flow fractionation procedures – or any immobilisation onto surfaces as is required by techniques such as surface plasmon resonance. It may well turn out that there may be no polysaccharide which gives an interaction that is strong enough – and resistant enough to external effects, but at least there is now another methodology to explore the interactions.

## Abbreviations

DF: Dietary fibre; Food and Agriculture Organisation of the United Nations; GF: Gluten-free; GFD: Gluten-free diet; HMW: High molecular weight; LMW: Low molecular weight; NDC: Non-digestible carbohydrate; S: Svedberg unit = 10^-13^sec; *s*: Sedimentation coefficient; *s*_*20*,w_: Sedimentation coefficient normalized to standard solvent conditions of the density and viscosity of water at 20^o^C; *s*^*o*^_*20*,w_: Sedimentation coefficient normalized to standard solvent conditions of the density and viscosity of water at 20^o^C and extrapolated to zero concentration to eliminate effects of non-ideality; WHO: World Health Organisation; Amino: Acids E- glutamic acid; F: Phenylalanine; G: Glycine; L: Leucine; P: Proline; Q: Glutamine; S: Serine; T: Threonine; X: Unknown/unspecified; Y: Tyrosine.

## Competing interests

The authors declare that they have no competing interests.

## References

[B1] Koning F Celiac disease: caught between a rock and a hard place Gastroent 2005 129 1294 1301 10.1053/j.gastro.2005.07.030 16230082

[B2] Marsh MN Transglutaminase, gluten and coeliac disease: Food for thought Nature Med 1997 3 725 726 10.1038/nm0797-725 9212095

[B3] Dicke WK Coeliakie: een onderzoek naar de nadelige invloed van sommige graansoorted op de lijder aan coeliakie (Investigation of the harmful effects of certain types of cereal on patients suffering from coeliac disease) 1950 University of Utrecht: MD Thesis

[B4] van de Kamer JH Weijers HA Coeliac disease. V. Some experiments on the cause of the harmful effect of wheat gliadin Acta Paediatr 1955 44 465 469 10.1111/j.1651-2227.1955.tb04269.x 13292281

[B5] Mulder CJJ Cellier C Coeliac disease: changing views Best Prac Res Clin Gastroent 2005 19 313 321 10.1016/j.bpg.2005.01.006 15925838

[B6] West J Logan RFA Smith CJ Hubbard RB Card TC Malignancy and mortality in people with coeliac disease: population based cohort stud BMJ 2004 329 716 10.1136/bmj.38169.486701.7C 15269095 PMC518895

[B7] Perrotta T The diet that shook up tennis? Wall Street J 2011 via online.wsj.com

[B8] Green P Jabri B Coeliac disease Lancet 2003 362 383 391 10.1016/S0140-6736(03)14027-5 12907013

[B9] Vader W Kooy Y van Veelen P de Ru A Harris D Benckhuijsen W Peña S Mearin L Drijfhout JW Koning F The gluten response in children with celiac disease is directed toward multiple gliadin and glutenin peptides Gastroent 2002 122 1729 1737 10.1053/gast.2002.33606 12055577

[B10] Williams A Stirling L Dietary compliance and long term follow-up of coeliac children in the East Midlands J Hum Nutr Dietet 2002 15 456

[B11] Periolo N Cherñavsky AC Coeliac disease Autoimmun Rev 2002 5 202 208 16483920 10.1016/j.autrev.2005.06.013

[B12] Gallagher E Gormley TR Arendt EK Recent advances in the formulation of gluten-free cereal-based products Trends Food Sci Tech 2004 15 143 152 10.1016/j.tifs.2003.09.012

[B13] Marconi E Careca M Pasta from non-traditional raw materials Cereal Foods World 2001 46 522 530

[B14] Collin P Mäki M Kaukinen K Safe gluten threshold for patients with coeliac disease: some patients are more tolerant than others Am J Clin Nutr 2007 86 260 17616789 10.1093/ajcn/86.1.260

[B15] Codex Alimentarius Standard for Special Dietary Use for Persons Intolerant to Gluten 1981; Revised 2008 Codex Standard 118

[B16] Olexova L Dovicovicova L Svec M Siekel P Kuchta T Detection of gluten-containing cereals in flours and “gluten-free” bakery products by polymerase chain reaction Food Control 2006 17 234 237 10.1016/j.foodcont.2004.10.009

[B17] Grehn S Fridell K Lilliecreutz M Hallert C Dietary habits of Swedish adult coeliac patients treated by a gluten-free diet for 10 years Scand J Nutrition 2001 45 178 182

[B18] Shewry PR Tatham AS The prolamin storage proteins of cereal seeds - structure and evolution Biochem J 1990 267 1 12 2183790 10.1042/bj2670001 PMC1131235

[B19] Piston F Dorado G Martín A Barroet F Cloning of nine gamma-gliadin mRNAs (cDNAs) from wheat and the molecular characterization of comparative transcript levels of gamma-gliadin subclasses J Cer Sci 2006 43 120 128 10.1016/j.jcs.2005.07.002

[B20] Ang S Kogulanathan J Morris GA Kök MS Shewry PR Tatham AS Adams GG Rowe AJ Harding SE Structure and heterogeneity of gliadin: a hydrodynamic evaluation Eur Biophys J 2010 39 255 261 10.1007/s00249-009-0529-7 19669133

[B21] Shewry PR Napier JA Tatham AS Seed storage proteins - structures and biosynthesis Plant Cell 1995 7 945 956 7640527 10.1105/tpc.7.7.945 PMC160892

[B22] Shimoni Y Galili G Intramolecular disulfide bonds between conserved cysteines in wheat gliadins control their deposition into protein bodies J Biol Chem 1996 271 18869 18874 10.1074/jbc.271.31.18869 8702547

[B23] Martucci S Biagi F Di Sabatino A Corazza GR Coeliac disease Digest Liver Dis 2002 34 150 153 10.1016/s1590-8658(02)80184-0 12408460

[B24] Anderson RP Degano P Godkin AJ Jewell DP Hill AVS In vivo antigen challenge in celiac disease identifies a single transglutaminase-modified peptide as the dominant A-gliadin T-cell epitope Nature Med 2000 6 337 342 10.1038/73200 10700238

[B25] Shan L Molberg Ø Parrot I Hausch F Filiz F Gray G Sollid LM Khosla C Structural basis for gluten intolerance in celiac sprue Science 2000 297 5590 2275 2279 12351792 10.1126/science.1074129

[B26] Tollefsen S Arentz-Hansen H Fleckenstein B Molberg Ø Ráki M Kwok WW Jung G Lundin KEA Sollid LM HLA-DQ2 and -DQ8 signatures of gluten T cell epitopes in celiac disease J Clin Invest 2006 116 2226 2236 10.1172/JCI27620 16878175 PMC1518792

[B27] Cabrera-Chávez F Islas-Rubio AR Rouzaud-Sández O Sotelo-Cruz N Calderón de la Barca AM Modification of gluten by methionine binding to prepare wheat bread with reduced reactivity to serum IgA of celiac disease patients J Cer Sci 2010 52 310 313 10.1016/j.jcs.2010.06.013

[B28] Trowell H Southgate DAT Wolever TMS Leeds AR Gassull MA Jenkins DJA Dietary fibre redefined Lancet 1976 1 967 57372 10.1016/s0140-6736(76)92750-1

[B29] Marsh MN Gluten, major histocompatibility complex and the small intestine Gastroent 1992 102 330 354 1727768

[B30] Byrnes AE Ellis PR Hartley LP Frost GS Novel functional bread reduces postprandial insulin resistance in healthy middle aged men at risk of coronary heart disease (CHD) over a twenty-four hour period J Hum Nutr Dietet 2002 15 462 469

[B31] Slavin JL Greenberg NA Partially hydrolyzed guar gum: Clinical nutrition uses Nutrition 2003 19 549 552 10.1016/S0899-9007(02)01032-8 12781858

[B32] Morris GA Castile J Smith A Adams GG Harding SE Macromolecular conformation of chitosan in dilute solution: A new global hydrodynamic approach Carbohyd Polym 2009 76 616 621 10.1016/j.carbpol.2008.11.025

[B33] Tolstoguzov VB Schwenke KD, Mothes R Functional properties of food proteins. Role of interactions in protein systems In Food Proteins ~~ Structure and Functionality (4th Symposium on Food Proteins “Structure-Functionality Relationships”, Reinhardsbrunn, Germany, 5–8 October, 1992) 1993 Weinheim u.a: Verlag Chemie 203

[B34] Harding SE Rowe AJ Insight into protein-protein interactions from analytical ultracentrifugation Biochem Soc Trans 2010 38 901 907 10.1042/BST0380901 20658974

[B35] Harding SE Trends in mucoadhesive analysis Trends Food Sci Tech 2006 17 255 262 10.1016/j.tifs.2005.12.007

[B36] Heinze T Nikolajski M Daus S Besong TMD Michaelis N Berlin P Morris GA Rowe AJ Harding SE Protein-like oligomerisation of carbohydrates Angew Chem Int Ed 2011 50 8602 8604 10.1002/anie.201103026 21786375

[B37] Song Y Gao L Li L Zheng Q Influence of gliadins on rheology of methylcellulose in 70 % (v/v) aqueous ethanol Food Hydrocolloid 2010 24 98 104 10.1016/j.foodhyd.2009.08.010

[B38] Seifert A Heinevetter L Colfen H Harding SE Characterization of gliadin-galactomannan incubation mixtures by analytical ultracentrifugation. Part I. Sedimentation velocity Carbohyd Polym 1995 28 325 332 10.1016/0144-8617(96)00004-5

[B39] Bemiller JN Whistler RL Fennema OR Carbohydrates Food Chemistry 1996 Third Ed New York: Marcel Dekker 157 223

[B40] Konig E Renner EJ Die Milcheiweipallergie: Ursuchan, Diagnose, Behandlung In Milchwissenschaft Giessen 1993 Liebig-Universität 15

[B41] Pinier M Verdu EF Nasser-Eddine M David CS Vézina A Rivard N Leroux J-C Polymeric binders suppress gliadin-induced toxicity in the intestinal epithelium Gastroent 2009 136 288 298 10.1053/j.gastro.2008.09.016 18992747

[B42] Harding SE Winzor DJ Harding SE, Chowdhry BZ Sedimentation velocity analytical ultracentrifugation Protein-Ligand Interactions: Hydrodynamics and Calorimetry 2001 Oxford University Press 75 103

[B43] Ang S Hydrodynamic Studies on Polysaccharides and their Interactions 2009 University of Nottingham: PhD Dissertation Chapter 8

